# Supplementary material for: Dynalign II: common secondary structure prediction for RNA homologs with domain insertions
Source: Nucleic Acids Res. 2014 Nov 21;42(22):13939–48. doi: 10.1093/nar/gku1172 (PMC4267632; doi:10.1093/nar/gku1172)
Supplement: SUPPLEMENTARY DATA [file supp_gku1172_nar-02021-z-2014-File012.zip › manual/javadoc/comments/InterfaceSource.html]

A package that encapsulates all the Java classes that comprise the RNAstructure Java interface.
  
  
There are no classes in this package.
Instead, all Java interface classes are in the subpackages of this one.
This is because the interface is backed by C++ classes, and these C++ classes are separated from the Java classes in the RNAstructure\_java\_interface folder.
C++ classes cannot be documented with Javadocs, but are documented with Doxygen formatting.
